# Your Flaws Are My Pain: Linking Empathy To Vicarious Embarrassment

**DOI:** 10.1371/journal.pone.0018675

**Published:** 2011-04-13

**Authors:** Sören Krach, Jan Christopher Cohrs, Nicole Cruz de Echeverría Loebell, Tilo Kircher, Jens Sommer, Andreas Jansen, Frieder Michel Paulus

**Affiliations:** 1 Department of Psychiatry and Psychotherapy, Philipps-University Marburg, Marburg, Germany; 2 Department of Neurology, Philipps-University Marburg, Marburg, Germany; 3 School of Psychology, Queen's University Belfast, Belfast, United Kingdom; 4 Department of Psychology, Philipps-University Marburg, Marburg, Germany; Kyushu University, Japan

## Abstract

People vicariously experience embarrassment when observing others' public pratfalls or etiquette violations. In two consecutive studies we investigated the subjective experience and the neural correlates of vicarious embarrassment for others in a broad range of situations. We demonstrated, first, that vicarious embarrassment was experienced regardless of whether the observed protagonist acted accidentally or intentionally and was aware or unaware that he/she was in an embarrassing situation. Second, using functional magnetic resonance imaging (fMRI), we showed that the anterior cingulate cortex and the left anterior insula, two cortical structures typically involved in vicarious feelings of others' pain, are also strongly implicated in experiencing the ‘social pain’ for others' flaws and pratfalls. This holds true even for situations that engage protagonists not aware of their current predicament. Importantly, the activity in the anterior cingulate cortex and the left anterior insula positively correlated with individual differences in trait empathy. The present findings establish the empathic process as a fundamental prerequisite for vicarious embarrassment experiences, thus connecting affect and cognition to interpersonal processes.

“When we are living with people who have a delicate sense of propriety, we are in misery on their account when anything unbecoming is committed. So I always feel *for* and *with* Charlotte when a person is tipping his chair. She cannot endure it.” [*Elective Affinities, J. W. Goethe*].

## Introduction

Imagine the following anecdotal situation: You are attending a conference. While sitting within a fully occupied audience you observe the presenter walking down the aisle with toilet paper clinging to his back pocket. Before you could take charge of the situation and make the presenter aware of the unwanted attention from everybody, you would be sure to imagine what others must think of him and what the source of giggling or averting the gaze would be. The presenter, on the other hand, would walk to the podium, unaware of why everybody is secretly looking at his backside. Despite the presenter's unspecific emotional state, surrounding bystanders experience strong emotions – vicariously. It is these vicariously experienced emotions and their neural underpinnings in response to intentionally or accidentally caused public shortcomings, pratfalls, or norm violations of others that are the focus of the present research.

Social emotions such as embarrassment, guilt, pride, or shame represent key elements of our human moral apparatus. They have been distinguished from basic emotions (fear, happiness, etc.) in various ways [1]. Recent conceptualizations suggest that embarrassment is a transient reaction to a violation of social etiquette that endangers one's particular public image and can be evoked in different situations. Examples include physical pratfalls (e.g., slipping in the mud), cognitive shortcomings, loss of control over the body, shortcomings in physical appearance (e.g., zipper open), or failure at privacy regulation [1], [2], [3].

Returning to the above anecdote, embarrassment is also experienced vicariously. Research shows that vicarious embarrassment is evoked even without any connection between the observer and the protagonist's predicament and without any responsibility of the observer for the protagonist's situation [4], [5], [6]. In his classic work Miller [4] hypothesized that maintaining face in social interactions is of such central concern that envisioning oneself in the place of an embarrassed other might cause one to suffer empathic embarrassment (p. 9).

### Neural Correlates of Empathy for Others' Pain

To date, research on the neural correlates of empathic processes has primarily focused on the empathic processing of others' physical pain. Functional magnetic resonance imaging (fMRI) studies report that affective-motivational components of the so called ‘pain matrix’ [7], [8], [9], the anterior insula and the anterior cingulate cortex (ACC), are most reliably involved in the compassionate feeling of pain [10], [11], [12], [13], [14], [15], [16], [17]. Further, subjective reports on the experienced pain as well as participants' estimates of the intensity of observed pain were positively associated with the hemodynamic responses in these regions [12], [16], [18]. More recently, an fMRI study extended these findings and demonstrated that compassion for both physical (i.e. injury) and social pain (i.e. social rejection) are processed in the anterior insula [19]. Based on these studies, the ACC and the anterior insula have been regarded as core regions involved in empathically processing the emotions of others and generating inner states of another person's feelings [20].

While concentrating on basic emotions, social neuroscience has not yet focused on the neural correlates of vicarious embarrassment experienced for others' flaws. However, some fMRI studies have investigated related concepts, such as the first-person experience of embarrassment [21] or the impact of intentional and accidental norm violations on inferred emotions using written vignettes [22], [23]. These studies asked participants to infer their own emotions imagining being the protagonist or estimating the emotion of the protagonist, respectively. These studies are thus limited in understanding the processes involved in vicarious embarrassment experiences.In line with the model of Miller (1987), we hypothesize that empathy processes provide the foundation to understand the complex emotion of vicarious embarrassment and postulate that individual differences in empathy modulate the embarrassment individuals experience for others' predicaments. Therefore, the observation of vicariously embarrassing situations should not only stimulate brain regions typically involved in empathic processes, but moreover individual differences in trait empathy should correlate with these neural activations.

### Modeling Intention Attribution and Perspective Taking in Vicarious Embarrassment

Previously used experimental procedures limit the examination of vicarious embarrassment experiences to situations that engage protagonists who are aware about their current transgression [6]. To better approximate the broader variety of situations that make observers experience vicarious embarrassment we propose a different approach in examining this social emotion. As for the first-person experience of embarrassment, an essential requirement for vicarious embarrassment is that the observed protagonist violates normative social standards or etiquettes. In contrast to the first-person experience of embarrassment, however, the norm-violating nature of the behavior does not need to be accessible to the protagonist; it is the observer who needs to be aware of it. Extending Miller's (1987) work, we postulate that it is an empathic process and, accordingly, the capability to represent other people's inner states that enables observers to experience vicarious embarrassment. However, when observers perceive somebody as violating a social etiquette in a public situation, they may not only put their self into the ‘mental shoes’ of the observed protagonist, his/her intentions and feelings, but also take into account their external view on normative and social standards in the particular context [24]. This process is far from perfect and rather egocentric than focused merely on the feelings of the observed person [25], and may account for situations that evoke vicarious embarrassment in observers even when the fact of the norm violating incident is not accessible to the observed protagonist himself/herself.

Drawing on research on the (non-vicarious) experience of embarrassment [2], [26], we further suggest that the attributed intentional or accidental character of a norm violation is crucial to the experience of vicarious embarrassment. Accidental mishaps do not necessarily reflect on one's personal character or flawed aspects of the self because the behavior can well be caused by external, situational factors. In contrast, attribution of intentionality to the protagonist's misbehavior directly reflects on his/her character. Here, the protagonist has control about his/her action and, accordingly, can be made responsible for his/her behavior. This distinction seems to be implicitly present in the existing empirical studies on vicarious embarrassment; however, it has not yet been integrated into a conceptualization of situations eliciting vicarious embarrassment.

The above considerations led us to organize situations in which vicarious embarrassment may occur along two dimensions. The dimension of “*intentionality*” represents the accidental versus intentional character of the embarrassing situation. The dimension of “*awareness*” refers to the accessibility of the norm violation to the observed protagonist. The dimensions are thought to be conceptually orthogonal, so their combination results in four distinctive classes of situations in which observers may experience vicarious embarrassment, abbreviated in the following as (i) **AA** (accidental ∩ aware); (ii) **AU** (accidental ∩ unaware); (iii) **IA** (intentional ∩ aware) and (iv) **IU** (intentional ∩ unaware).

### Overview of the Present Studies

The present research aims to elucidate the phenomenon of vicarious embarrassment, its link to individual differences in trait empathy, and the underlying neuro-cognitive processes. In two pilot studies, we constructed and validated stimulus situations to elicit vicarious embarrassment representing the hypothesized dimensions of “*intentionality*” and “*awareness*” (see File S1). In Study 1, we showed that vicarious embarrassment is experienced independently of the experience of first-person embarrassment and that vicarious embarrassment is related to individual differences in trait empathy. In Study 2, we used functional magnetic resonance imaging (fMRI) to transfer this modulation of individual differences in trait empathy to the level of neural activations during the processing of vicariously embarrassing situations. We discuss these findings in the light of contemporary research on empathy for physical pain.

## Study 1: Relation of Vicarious Embarrassment to First-Person Embarrassment and Individual Differences in Empathy

First, we expected that vicarious embarrassment experiences would differ in their experience from the first-person embarrassment as attributed to an observed person in a specific situation. On the one hand, as previous research suggests, vicarious embarrassment may depend on the observation of embarrassment in others. These situations are characterized by a protagonist accidentally being exposed in a public predicament that he/she is aware of. On the other hand, as suggested above, for a broad range of other situations we expect that vicarious embarrassment is independent of the perception of embarrassment in others. These situations comprise others' intentional norm violations or the protagonists being unaware about the embarrassing incident. Second, we examined the role of individual differences in trait empathy in experiencing vicarious embarrassment. With processes of perspective taking considered at the core of this emotional reaction [4], we assumed that trait empathy would be positively related to the self-reported experience of vicarious embarrassment.

### Methods

#### Ethics Statement

We confirm that the research has been conducted in compliance with the appropriate ethical guidelines of the American Psychological Association (APA). The study was approved by the local ethics committee at the faculty of medicine, Philipps-University Marburg. All subjects were written informed about the background of the study and anonymity of data collection. We confirm that we obtained informed written consent from all participants involved in the study.

#### Participants

The sample consisted of 480 women and 139 men, all German speaking, with a mean age of 23.79 (*SD* = 3.66) years. A majority of 534 participants (86.3%) were undergraduate students, 37 (6.0%) indicated they were unemployed, 32 (5.2%) worked or received professional training, and 14 were pupils (2.3%). Twelve participants did not specify their educational or professional status.

#### Materials and Procedure

Fifty-two previously selected vignettes modeling the four different situations (see File S1) were used in an online questionnaire (www.limesurvey.org; version 1.72). All participants started with the questionnaire (including two exemplary vignettes in the introduction) asking to evaluate the embarrassment reaction for each of the 52 vignettes as they thought the observed acting person would experience at that specific moment: *“Imagine yourself observing the following situation. What do you think: Is the* ***person*** *feeling embarrassed at that specific moment? If yes, how intense is this feeling?”* The relevant protagonist was highlighted in bold. After this, participants received the vicarious embarrassment instruction and rated their vicarious embarrassment experiences for each of the situation vignettes. The instructions were similar to those in the pilot studies: “*Imagine* ***you*** *are observing the person in the situation. Are* ***you*** *feeling embarrassed for this person? If yes, how intense is this feeling?*”. Responses were given on scales ranging from 1 (*not at all*) to 7 (*very strong*). The relevant protagonist was highlighted in bold to clarify the perspective the participants were asked to take. The order of block presentation (i.e. first embarrassment then vicarious embarrassment) was chosen to minimize effects that vicarious embarrassment ratings could exert on embarrassment ratings. The stimulus material varied regarding the underlying dimensions of “*intentionality*” and “*awareness*” and clustered into four distinctive categories. Eleven neutral situations displaying appropriate behavior complemented the set of stimuli.

#### Accidental ∩ Aware (AA, 11 vignettes)

The predicament occurs accidentally while the protagonist is fully aware of the inappropriateness of his or her behavior. Examples include stumbling during a speech and slipping in the mud.

#### Accidental ∩ Unaware (AU, 10 vignettes)

Likewise, the predicament occurs accidentally, but the protagonist does not realize the mishap within that specific moment as the incident is out of his or her attentional or perceptual focus. Examples include walking around with the zipper open and, as exemplified in the introduction, having toilet paper hanging out of the back pocket.

#### Intentional ∩ Aware (IA, 10 vignettes)

Here, the norm violation is intentionally evoked, although the protagonist is well aware that his/her behavior is inappropriate in the current situation. Examples include belching aloud in a high-end restaurant and throwing garbage on the street.

#### Intentional ∩ Unaware (IU, 10 vignettes)

Finally, certain conventions are violated without the protagonist being aware about the inappropriateness underlying that particular situation. Examples include extensive self-praising in public speeches or wearing a T-shirt with an imprint stating ‘I am sexy’.

After the vicarious embarrassment ratings, participants received instructions for completing the E-Scale [27] (see File S2). The E-scale measures individual difference in trait empathy with 25 items, representing a general empathy factor. The items cover different empathic behaviors and participants rate how well these behaviors apply to themselves on a scale from 1 (*not at all*) to 5 (*very strong*). It has been shown that the scale discriminates between the two related sub-facets of “emotional” and “cognitive” empathy [27]. In line with conceptualizations of trait empathy as a multi-dimensional construct [27], [28], the emotional facet of the E-Scale describes individual differences in the experience of another's emotion, whereas the cognitive facet focuses on the mental act to take another's perspective. Example items include “I can easily relate to the feelings of literary characters” (emotional empathy) and “I sometimes try to better understand my friends by taking their perspective” (cognitive empathy). The E-Scale shows good convergent and discriminant validity as well as internal consistency and re-test reliability [27]. In the present sample the overall E-Scale (α = .91) as well as the emotional (α = .82) and cognitive (α = .84) facets had high internal consistencies. At the end of the questionnaire, socio-demographic variables were assessed. The whole questionnaire took about 40 minutes to complete.

#### Data Analyses

Data were analyzed with PASW Statistics 18 (SPSS, 2009, Chicago, IL). Each participant's ratings were averaged across the situations in each category (AA, AU, IA, IU, and Neut). Internal consistencies for the different vicarious embarrassment situations were high (Cronbach's α first-person embarrassment: AA = .86; AU = .96; IA = .88; IU = .85; vicarious embarrassment: AA = .93; AU = .93; IA = .89; IU = .90). Averaged ratings (AA, AU, IA, and IU) were entered into a two-factorial repeated-measures General Linear Model (GLM) with the factor PERSPECTIVE (first-person embarrassment vs. vicarious embarrassment) and the factor CATEGORY (the four levels of different situations). Individual differences in empathy as measured by the E-Scale were entered as a continuous factor that was specified to interact with the other two factors. Three contrasts comparing the AA situations with each of the other three categories (1 -1 0 0; 1 0 -1 0; 1 0 0 -1) were implemented to test the corresponding simple and interaction effects. Descriptions of effect sizes are reported as partial eta-squares in case of significant effects. Differences to neutral situations were tested separately for each of the four categories with paired-sample t-tests in both conditions. To further elucidate the effects of individual differences in trait empathy on ratings of vicarious embarrassment and first-person embarrassment, Pearson correlations were computed separately for each category and the neutral situations.

### Results

Results of the GLM showed a significant main effect of CATEGORY, *F*(3, 1851)  = 47.61, *p*<.001, *η* ^2^ = .072, but not of PERSPECTIVE, *F*(1, 617)  = 2.03, *p* = .154, and a significant two-way interaction between CATEGORY and PERSPECTIVE, *F*(3, 1851)  = 39.10, *p*<.001, *η* ^2^ = .060 (all *ps* Greenhouse-Geisser corrected for non-sphericity). More specifically, the contrasts of AA vs. each of the other three categories revealed significant effects (AA vs. AU: *F*(1, 617)  = 84.03, *p*<.001, *η* ^2^ = .120; AA vs. IA: *F*(1, 617)  = 77.98, *p*<.001, *η* ^2^ = .112; AA vs. IU: *F*(1, 617)  = 72.89, *p*<.001, *η* ^2^ = .106). Importantly, the interaction of these contrasts with PERSPECTIVE were all significant (AA vs. AU: *F*(1, 617)  = 44.90, *p*<.001, *η* ^2^ = .068; AA vs. IA: *F*(1, 617)  = 78.47, *p*<.001, *η* ^2^ = .113; AA vs. IU: *F*(1, 617)  = 86.11, *p*<.001, *η* ^2^ = .122). Mean ratings of vicarious embarrassment for each class of situation were in the mid-range of the scale (see Table 1) and highest ratings were found for AA vignettes (*M* = 4.32, *SD* = 1.55) with continuously dropping magnitude toward IU (*M* = 3.28, *SD* = 1.42). A similar rank order was found for first-person embarrassment (*M* = 5.90, *SD* = 0.80 (AA); *M* = 1.39, *SD* = 0.60 (IU)), thus illustrating the main effect of CATEGORY found in the GLM. The interaction between CATEGORY and PERSPECTIVE was predominantly due to differences of AA situations from the other three categories (AU, IA, IU) as indicated by the contrast analyses. For situations in which the protagonist was aware of his or her accidental mishap (AA) attributed first-person embarrassment was stronger than the self-reported vicarious response of the participants (*d* = −1.28). For the other three types of situations the vicarious embarrassment experiences significantly exceeded the first-person embarrassment as attributed to the protagonist (*d* = 1.00 to 1.73, with all lower bounds of the confidence intervals exceeding *d* = 0.80). Paired-sample t-tests showed significant differences of each category (AA, AU, IA, IU) from neutral situations for both levels at PERSPECTIVE, all *T*s(618) >14.73, *p*<.001. Table 1 summarizes the results for first-person embarrassment as attributed to the protagonist and vicarious embarrassment ratings as indicated by the ‘observing’ participants.

**Table 1:** Vicarious embarrassment and first-person embarrassment across categories (Study 1).

	First-Person Embarrassment			Vicarious Embarrassment				95% CI		
	M	(SD)	Median	M	(SD)	Median	d-value	lower		upper
AA	5.90	(0.80)	6.09	4.32	(1.55)	4.45	-1.28	-1.41	;	-1.15
AU	2.35	(1.56)	1.60	3.86	(1.46)	3.90	1.00	0.89	;	1.11
IA	2.11	(0.94)	1.90	3.73	(1.38)	3.70	1.37	1.24	;	1.51
IU	1.39	(0.60)	1.20	3.28	(1.42)	3.20	1.73	1.58	;	1.89
Neutral	1.05	(0.14)	1.00	1.02	(0.08)	1.00	-0.26	-0.34	;	-0.18

*Note.* Responses were given on scales ranging from 1 (*not at all*) to 7 (*very strong*). Positive *d*-Values indicate higher ratings under the vicarious embarrassment condition. Situations are abbreviated based on the intentionality and the belief state of the observed protagonist in the vicarious embarrassing situation. AA  =  Accidental ∩ Aware, AU  =  Accidental ∩ Unaware, IA  =  Intentional ∩ Aware, IU  =  Intentional ∩ Unaware.

Individual differences in trait empathy had a significant impact on ratings, *F*(1,617)  = 42.98, *p*<.001, *η* ^2^ = .065. The interactions of empathy with CATEGORY, *F*(3,1851)  = 8.09; *p*<.001; *η* ^2^ = .013 and PERSPECTIVE, *F*(1,617)  = 21.16, *p*<.001, *η* ^2^ = .033 were significant, but the three-way interaction between CATEGORY, PERSPECTIVE, and trait empathy did not reach significance, *F*(3,1851)  = 0.361, *p* = .746. To further examine the interactions involving trait empathy, Table 2 provides correlation coefficients of individual differences in trait empathy with first-person embarrassment and vicarious embarrassment ratings. Overall, empathy was positively related to ratings at both perspectives, illustrating the main effect of the empathy in the GLM. However, the strongest correlations emerged with vicarious embarrassment ratings. Correlations were medium to small in size, ranging from *r* = .20 to *r* = .24 (AA), *r* = .24 to *r* = .28 (AU), *r* = .19 to *r* = .23 (IA), and *r* = .13 to *r* = .16 (IU). Emotional and cognitive facets of trait empathy showed similar associations with vicarious embarrassment ratings (see Table 2). As indicated by the significant interaction between PERSPECTIVE and empathy, the ratings of first-person embarrassment as attributed to the protagonist showed weaker associations with individual differences in trait empathy. Significant correlations were found in AA situations (ranging from *r* = .14 to *r* = .21) which together with the vicarious embarrassment ratings in AA situation illustrated the interaction of empathy with CATEGORY in the GLM. Ratings for neutral scenarios did not substantially correlate with individual differences in trait empathy (.05≥ *r* ≥ −.08).

**Table 2:** Correlations of first-person embarrassment and vicarious embarrassment ratings with individual differences in trait empathy (Study 1).

		First-Person Embarrassment					Vicarious Embarrassment				
		AA	AU	IA	IU	Neut	AA	AU	IA	IU	Neut
Empathy		**.19**	.10	.10	.07	.02	**.24**	**.28**	**.22**	**.16**	-.05
	Emotional	**.14**	.06	.05	.04	.00	**.20**	**.24**	**.19**	**.13**	-.08
	Cognitive	**.21**	**.14**	**.13**	.08	.05	**.23**	**.28**	**.23**	**.16**	-.01

*Note. N*  =  619. Statistically significant correlations at *p*<.001 are printed in bold. AA  =  Accidental ∩ Aware, AU  =  Accidental ∩ Unaware, IA  =  Intentional ∩ Aware, IU  =  Intentional ∩ Unaware, Neut  =  Neutral.

#### Discussion Study 1

Study 1, using written stimulus material in a within-participants design, demonstrates that observers' vicarious embarrassment does not necessarily require observing embarrassment in others. Among the four categories of vicariously embarrassing situations, only in AA situations did the emotional reaction as attributed to the awkwardly behaving person exceed the vicarious embarrassment experiences in observers. However, following our prediction about the variety of situations eliciting vicarious embarrassment, self-reported vicarious emotions substantially outweighed the first-person embarrassment attributed to the protagonist. This was shown in case of situations where the protagonist was (physically) unaware of his/her violation of a social etiquette (AU) or intentionally violated a social norm, whether aware of that fact or not (IA and IU).

Further, individual differences in trait empathy were positively correlated with vicarious embarrassment ratings across all situations. The effects are notable for several reasons. First, correlations of trait empathy were higher with vicarious embarrassment ratings than with ratings of first-person embarrassment or in neutral situations. This indicates that the association of trait empathy and vicarious embarrassment cannot be explained by the attribution of embarrassment experiences to others. In the present data this process might occur in situations in which one observes embarrassment in others (AA), but it does not generalize to situations where the protagonists themselves are not aware about the embarrassing incident (AU, IA, IU). Second, the correlations found in this study provide additional insight into the conceptualization of empathy as a co-experience of an actor's personal state [27], [28]. It is inherent in the concept that highly empathic individuals are more strongly influenced by others' distress and emotions in their personal experience than individuals low in empathy (AA) [27]. However, to our knowledge, this is the first study to show empirically that vicarious social emotions, here embarrassment, are related to individual differences in empathy without sharing an emotional condition (AU, IA, IU).

Intriguing at first glance, this finding may be due to the fact that taking the perspective and sharing of another person's inner state is influenced by additional information that is accessible to the observer in a social situation [24], [25]. The construal of another's state is thus egocentrically biased by one's own stereotypes, ideology, prior knowledge, and other idiosyncratic information [24]. Even when people are fully aware that another's perspective differs from their egocentric assessment of the situation, it is one's own perspective that is thought to serve as an anchor. For psychotherapists it is of high relevance to overcome this egocentrism, however, this does not apply for spontaneous reactions in everyday life situations as captured in the vicarious embarrassment situations. Accordingly, a recent study showed that vicarious embarrassment experiences increase when imagining oneself in an observed embarrassing situation compared to being the protagonist [29]. This reasoning explains why people experience vicarious embarrassment for a protagonist who is currently not experiencing embarrassment at his or hers own flaws and why individual differences in trait empathy are positively correlated with this experience. However, the observed correlations were not very high. Thus, substantial unshared variance indicates that other processes related to the idiosyncratic evaluation of the situation are involved. This establishes vicarious embarrassment as a concept that is clearly separate from classic definitions of empathy and suggests the distinction of forms of empathy that are more or less a co-experience of another's emotion, and forms of empathy that rather reflect the observer's evaluation of the situation in the social context.

## Study 2: The Neural Correlates of Vicarious Embarrassment and Their Link to Individual Differences in Empathy

Extending Study 1, we hypothesized that the perception of others' flaws or norm violations results in activations in brain regions commonly associated with empathic processes. Based on research on empathy for pain and basic emotions we hypothesized that vicarious embarrassment would result in stronger activations in the anterior insula and the ACC as neural indicators for the ongoing empathic process [13], [17]. Moreover, if empathy is considered crucial in conceptualizing vicarious embarrassment, the anterior insula as well as the ACC should show strong hemodynamic responses even for situations where the protagonist does not experience any social emotion him-/herself (i.e. AU, IA, IU). We expected that individual differences in trait empathy would be positively related to the activation of these two structures while observing vicariously embarrassing situations.

### Methods

#### Ethics Statement

We confirm that the research has been conducted in compliance with the appropriate ethical guidelines of the American Psychological Association (APA). The study was approved by the local ethics committee at the faculty of medicine, Philipps-University Marburg. All subjects were written informed about the background of the study and anonymity of data collection. We confirm that we obtained informed written consent from all participants involved in the study.

#### Participants and Data Acquisition

Thirty-two right-handed subjects (Philipps-University Marburg undergraduate students, 17 female, aged 20-28 years, *M* = 22.81, *SD* = 2.19) participated in the fMRI study for payment. The inclusion criteria were age (18-30 years) and absence of any psychiatric or neurologic disorder according to ICD-10. All subjects were native German speakers and had normal or corrected-to-normal vision. Participants were scanned at 3T (Siemens Trio, Erlangen) with 36 near-axial slices and a distance factor of 10% providing whole brain coverage. An echo planar imaging (EPI) sequence was used for acquisition of 553 functional volumes during the experiment (TR = 2.2 s, TE = 30 ms, flip angle = 90°, slice thickness = 3 mm, FoV = 192). After scanning, participants completed the E-Scale (Cronbach's α = .88) to measure individual differences in trait empathy and were debriefed afterwards.

#### Stimulus Material

**Figure 1:**
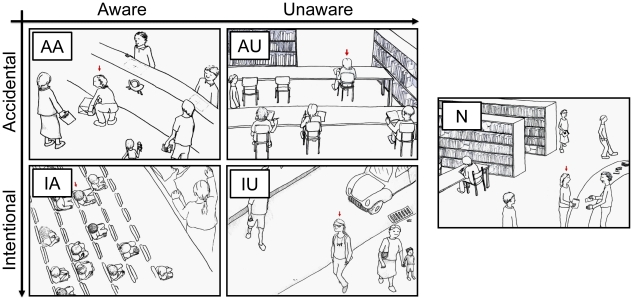
Examples of the stimulus material applied in the fMRI study. Drawn sketches depict a protagonist, indicated by the red arrow above his/her head, in possibly embarrassing situations. During the fMRI experiment each sketch was accompanied by a sentence introducing the current scenario. Situations were presented with the following textual vignettes for clarification: **AA**: You are at a post-office: the trousers of a person in front of you rip as he bends down to lift a package…; **AU**: You are in a library: the person in front of you wears her pants in a way that you can see her slip…; **IA**: You are at a cinema: during the movie a person in the front row is talking on his mobile…; **IU**: You are in a pedestrian zone: a young man wearing a VIP-necklet passes by…; **N**: You are in a library: a woman is borrowing a book at the reception desk…

The stimulus material consisted of a set of 50 sketches which were drawn based on a subset of situations that were examined in Study 1 (see Fig. 1; for detailed information see File S3). Sketches were used in addition to written vignettes to generate more potent stimuli for the fMRI experiment. For each modeled category (i.e. AA, AU, IA, IU) as well as for the neutral control situations ten sketches depicting a protagonist in a social situation were used.

#### Functional MRI Paradigm

All sketches were presented for 12 s together with a two-sentence description of the situation similar to Study 1. The text was presented in a black 24-point non-serif font (Arial) on a white background in two to three rows below the drawings. The stimulus presentation was followed by a blank screen for 1 s and a subsequent rating period lasting 3 s. During the rating period subjects were asked to indicate their level of vicarious embarrassment experienced during the previous picture story. Responses were indicated on a scale ranging from 1 (*not at all*) to 5 (*very strong*) using a button press of the right hand. A jittered low-level baseline showing a fixation cross for an average of 8 s was interleaved between the rating phase and the following trial. Sketches were presented in a pseudo-randomized order, ensuring that no type of situation was immediately repeated and all types of situation appeared in equal numbers. The total experiment time was 22.28 min. Stimuli were presented on an LCD screen with Presentation 11.0 software package (Neurobehavioral Systems, Albany, CA, USA, http://www.neurobs.com/). Prior to the experiment participants received careful instructions about the experimental procedure outside the scanner using two example situations that were not displayed during the fMRI session.

#### Functional MRI Data Analysis

FMRI data were analyzed using SPM8 (www.fil.ion.ucl.ac.uk/spm). The first four volumes (dummy images) of the session were discarded from further analyses. The remaining 549 EPI volumes were motion-corrected and spatially normalized to the standard template of the Montreal Neurological Institute (MNI). The normalized volumes were resliced with a voxel size of 2×2×2 mm, smoothed with an 8 mm full-width half-maximum isotropic Gaussian kernel and high-pass filtered at 1/256 Hz to remove low frequency drifts.

Statistical analysis was performed in a two-level, mixed-effects procedure. The fixed-effects GLM on the first level included six epoch regressors modeling hemodynamic responses to the vicariously embarrassing situations (4), neutral situations (1), and rating phase (1) with the abovementioned stimulus durations. The vicarious embarrassment ratings after each situation were entered as parametric modulators to explain additional variance in neural activation due to differences in emotional responses on the within-subject level. Six additional regressors modeling head movement parameters were introduced to account for noise. Individually weighted *ß*-maps of activation differences between the vicarious embarrassment and the neutral situations were analyzed on the second level.

The second-level analysis of activation differences was conducted with a full factorial random-effects GLM. The GLM contained one factor with the four dependent levels of vicarious embarrassment situations. The analysis of activation differences between vicariously embarrassing and neutral situations was controlled for individual differences in vicarious embarrassment experiences during the fMRI session by introducing subjects' averaged ratings within each type of situation as a covariate in the GLM. For the following analysis, brain regions with strongest associations with the different types of vicariously embarrassing situations were identified through a conjunction analysis of the contrasts of vicarious embarrassment compared to neutral situations (AA > Neutr ∩ AU > Neutr ∩ IA > Neutr ∩ IU > Neutr). The result of the conjunction-analysis was thresholded at *p*<.05 applying family-wise error (FWE) correction for whole brain analysis. All results are reported in MNI space.

#### Correlations with Individual Differences in Trait Empathy

**Figure 2:**
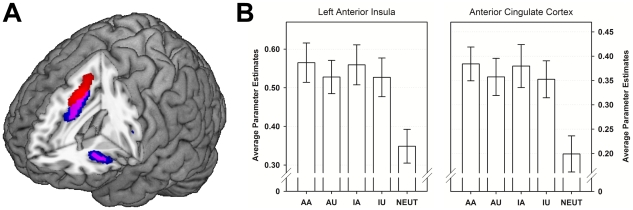
Neural activation and average parameter estimates during vicarious embarrassment. **A** Activations in response to vicarious embarrassing situations within the anterior cingulate cortex (ACC) and left anterior insula. The rendered image displays the results of a random-effects analysis contrasting vicarious embarrassing with neutral situations. Positive effects of a conjunction analysis (AA > N ∩ AU > N ∩ IA > N ∩ IU > N) thresholded at *p*<.05, FWE-corrected (red and pink areas) are superimposed on the regions-of-interests which were generated in the ACC and the anterior insula at a more liberal threshold, *p*<.001, uncorrected (blue and pink areas). **B** Average parameter estimates within the ACC and the left anterior insula masks during the processing of vicarious embarrassing (AA, AU, IA, IU) and neutral situations (NEUT).

To minimize circularity of the analyses estimates of brain activation were extracted independent of individual differences in trait empathy. First, functional regions-of-interest (ROIs) were constructed for the ACC and anterior insula. To achieve best estimates of the areas activated in vicariously embarrassment situations, the results of the conjunction analysis were thresholded at *p*<.001, uncorrected (see Figure 2A, blue and pink areas). To verify that the functional ROIs still correspond to the anatomical structures, these results were constrained by anatomical masks covering the insula and the middle/anterior cingulate cortex. The anatomical masks were created according to the anatomical labeling atlas implemented in the WFU-PickAtlas (Version 2.4) applying a dilation factor of one. Second, the averages of parameter estimates in the functionally and anatomically constrained clusters were computed for each individual. The resulting parameters for blood oxygenation level dependent (BOLD) responses in the ACC and the left insula were then entered into a repeated-measures GLM. The repeated factor CATEGORY covered the four levels of vicariously embarrassing situations, and individual differences in empathy were entered as a continuous factor. To further elucidate the effects of individual differences in trait empathy on ratings of vicarious embarrassment correlations were computed separately for each category and the emotional and cognitive subfacets of the E-scale.

### Results

#### Behavioral Data

The behavioral data of the fMRI study confirmed the findings of Study 1. As expected, participants rated their vicarious embarrassment significantly stronger during AA, AU, IA and IU situations compared to the neutral scenarios, *T*s(31) >12.87, *p*s<.001. Vicarious embarrassment ratings were around the scale mean with *M* = 2.46, *SD* = 0.64 (IU), *M* = 2.86, *SD* = 0.71 (IA), *M* = 3.21; *SD* = 0.79 (AU), and *M* = 3.50; *SD* = 0.70 (AA). Further, averaged vicarious embarrassment ratings differed significantly across the four modeled situations, *Ts*(31) >3.2, *p*s<.003.

Compared to the neutral situations (*M* = 786 ms, *SD* = 158 ms) participants had longer response times for their ratings after vicarious embarrassment situations (ranging from *M* = 995 ms, *SD* = 332 ms (IU) to *M* = 1039 ms, *SD* = 312 ms (AA); *t*s(31) >3.89, *ps*<.001). Reaction times did not significantly differ among the four types of situations, *T*s(31) <1.71, *ps*>.097. Pearson correlations of individual differences in trait empathy with vicarious embarrassment ratings were in a similar range as in Study 1 ranging from *r* = .17 (IA) to *r* = .32 (AA). However, at this sample size, trait empathy was only correlated significantly with ratings in AA situations (*p* = .036), not the other three situations (AU, IA, IU, *p*s>.051).

#### Neuroimaging Data

As predicted, the conjunction analysis revealed BOLD responses in brain areas typically involved in empathic processing to be significantly stronger for vicarious embarrassment situations than for neutral situations, *p*<.05, corrected (see Figure 2, Table 3). The activation of both the left ACC (−6 20 44) and the left anterior insula (-32 24 0) showed considerable overlap with the affective-motivational part of the ‘pain matrix’ [9], [20]. Furthermore we observed stronger responses in the thalamus, periaqueductal grey (PAG) in the brainstem, and the cerebellum, structures that are frequently associated with the empathic perceptions of others' pain [18].

**Table 3:** Common neural activations in response to vicarious embarrassing situations (Study 2).

	Side	MNI Coordinates			ClusteSize	T	p<
		x	y	z			
Anterior Cingulate Cortex	L	-6	20	44	395	6.75	.001
	L	-4	14	54		5.99	
	L	-10	28	28		5.66	
Anterior Insula	L	-32	24	0	62	5.66	.001
	L	-36	22	-16		4.19	
	L	-48	30	10		3.47	
Thalamus	L	-4	-6	6	23	5.33	.005
Cerebellum	R	30	-68	-28	5	4.98	.022
PAG	L	-2	-20	-22	1	4.84	.038

*Note. p*-values are reported at cluster-level and corrected for multiple comparisons in whole-brain analyses (FWE). Coordinates represent peak activations within a cluster. PAG  =  periaqueductal grey.

The GLMs with the parameters of the ACC and left anterior insula as dependent variables did not show significant effects of CATEGORY (ACC: *F*(3,90)  = 1.66; *p* = .181; anterior insula: *F*(3,90)  = 2.69; *p* = .061, Greenhouse-Geisser corrected). This was expected since the parameter estimates were extracted in regions showing the strongest activation in the conjunction analysis across the four situations. However, individual differences in trait empathy were significant predictors of the neural activation in both regions (ACC: *F*(1,30)  = 10.01, *p*<.005, *η* ^2^ = .250, anterior insula: *F*(1,30)  = 7.04, *p*<.013, *η* ^2^ = .190). The interaction of empathy with CATEGORY was non-significant for both the ACC and anterior insula indicating similar correlations of trait empathy with neural activation across all modeled situations (ACC: *F*(3,90)  = 1.59, *p* = .206; anterior insula: *F*(3,90)  = 2.69, *p* = .061). Further examination of the main effects found in the GLM indicated that BOLD responses of the anterior insula and the ACC were positively correlated with individual differences in trait empathy (see Table 4). Across the four types of situations the correlations of BOLD responses in the ACC with individual differences in trait empathy represented medium to large effects, varying from *r* = .33, *p* = .069 (AA) to *r* = .49, *p*<.002 (IA). Correlations with the subfacets emotional and cognitive empathy were in a similar range from *r* = .29, *p* = .111 (IA) to *r* = .49, *p*<.005 (IU). BOLD response of the left anterior insula showed similar correlations with individual differences in trait empathy ranging from *r* = .28, *p*<.121 (AA) to *r* = .50, *p*<.004 (IU) and correlations with the emotional and cognitive subfacets of empathy were between, *r* = .19, *p* = .302 (AU) and *r* = .52, *p*<.002 (IU).

**Table 4:** Correlations of average parameter estimates within the ACC and the left anterior insula with individual differences in trait empathy and vicarious embarrassment ratings.

		ACC				Left Anterior Insula			
		AA	AU	IA	IU	AA	AU	IA	IU
Empathy		**.33**	**.48**	**.49**	**.49**	.28	**.33**	**.49**	**.50**
	Emotional	.29	**.37**	**.49**	**.41**	.21	.19	**.47**	**.40**
	Cognitive	**.30**	**.48**	**.48**	**.48**	**.33**	**.42**	**.49**	**.52**
Ratings		.20	.21	.28	.23	**.45**	**.47**	**.33**	.26

*Note.* Correlations significant at *p*<.05 are printed in bold. ACC  =  Anterior Cingulate Cortex. AA  =  Accidental ∩ Aware, AU  =  Accidental ∩ Unaware, IA  =  Intentional ∩ Aware, IU  =  Intentional ∩ Unaware.

Participants' ratings of vicarious embarrassment experiences in each category showed positive associations with the neural activation, but these were statistically significant only for the left anterior insula. Correlations ranged from *r* = .26, *p* = .157 (IU) to *r* = .47, *p*<.006 (AU) in the anterior insula and from *r* = .20, *p* = .282 (AA) to *r* = .28, *p* = .126 (IA) in the ACC (see Table 4).

#### Discussion Study 2

The results of the fMRI study demonstrated that the observation of vicarious embarrassment situations elicit cortical activations in areas constituting the affective component of the pain matrix: the ACC and the left anterior insula [11], [12], [13], [14], [16], [17], [18], [30]. This activation was consistently high along a broad range of different situations and was even observed for situations which depicted the protagonist being unaware about the inappropriateness of the situation or intentionally violates social norms (AU, IA, and IU). Additionally, individual differences in vicarious embarrassment experiences during the fMRI session were positively correlated with the neural responses in the anterior insula.

Recent fMRI studies could show that the neural response to another person's physical pain was modulated by the observer's appraisals due to prior interactions with a protagonist [31] or own professional experience [32]. It was further demonstrated that even when observing photographs that display usually painful needle injections to an anesthetized hand, observers activate similar cortical areas [15]. In this line, the results of the present fMRI experiment show that in the absence of another’s embarrassment observers generate vicarious emotions based on their own appraisals. These appraisals depend on egocentric evaluations influenced by own stereotypes, attitudes, or assumptions about what is appropriate in a given social context. However, the correlations of individual differences in trait empathy with neural activation in the ACC and the anterior insula suggest that these appraisals are nevertheless embedded in processes of empathic perspective taking. Thus, vicarious embarrassment might in part result from the projection of oneself into the shoes of others observed in an inappropriate condition [24]. An analogy for perceiving potentially painful situations in others would be, for example, observing protagonists intentionally harming themselves (i.e. masochists; a suggestion brought forward by Lieberman [33]) or accidentally injuring themselves while not being aware of this fact (i.e. a paraplegic person clamping his or her feet in the wheelchair).

## Discussion

Worldwide millions of people gather in groups to watch television shows such as “Pop Idol” (United Kingdom), “America's Next Top Model” (United States), “Deutschland sucht den Superstar” (Germany), “Nouvelle Star” (France), or “Super Girl” (China) and collectively enjoy witnessing plights or mishaps happening to the candidates, and perceive “vicarious embarrassment”, “*Fremdscham*”, or “embarrassment-by-proxy”. The appeal of observing others' plights exploited via television or internet seems to be present regardless of whether the person in focus realizes the mishap (e.g., tripping, as “America's Next Top Model”) or not (e.g., singing with a bad voice, as a German “Superstar”). Although the effect of laughing about others' misfortunes has always been picked up in theater plays and comedy movies (e.g., early slapstick comedians such as Charlie Chaplin, Buster Keaton, or Laurel & Hardy exactly utilized this type of humor), today's media increasingly focuses on these everyday situations not only to laugh about but to feel *with* and *for* others to the entertainment of millions of spectators.

Current scientific approaches aiming to unravel the neuro-cognitive underpinnings of empathy for others' predicaments focus on the observation of physically painful scenarios, such as cutting one's fingers while cooking [10], [15], [18], [30]. Only recently, Yang and co-workers have demonstrated that the same cortical network implicated in empathically feeling (physical) pain is also involved in processes of compassion for others' social pain (i.e. states of social rejection [19]).

With the present study we extended these previous findings by showing that empathy for others' (social) pain – here embarrassment – is experienced in a broad range of different social situations and that inter-individual differences in empathy modulate the vicarious experience of embarrassment. Furthermore, we uncovered the neural correlates of the social emotion vicarious embarrassment and highlighted its link to individual differences in trait empathy.

We proposed the two orthogonal dimensions of “*intentionality*” and “*awareness*”, as attributed to the observed protagonist's actions, to classify situations into four distinctive categories. Using multimodal (written and pictorial) stimulus material we highlighted the importance of this approach for the understanding of the concept of vicarious embarrassment and its consequences. We consider two aspects as most relevant for the validity of the proposed dimensional structure. First, the relationship between first-person embarrassment and experienced vicarious embarrassment depends on situational characteristics; and second, behavioral as well as functional activity measures were found to correlate with individual differences in empathy.

With respect to first-person embarrassment in others, appeasement gestures are reasonably easy to decode via nonverbal channels. For example, controlled smiles, averted gaze, head movements down and away, lowered head, downcast eyes, diminished posture, and blushing have been proposed to constitute universally recognized gestures [1], [34], [35], [36], [37], [38]. Such gestures serve as a signal from empathic observers that the plight they have witnessed is an exceptional occurrence for the protagonist. This suggests that the apparent experience of embarrassment following a public blunder serves a social function. It reassures the observer that the protagonist recognizes that some etiquette has been violated, and it may therefore lead observers to view the protagonist more positively than would otherwise be the case [39], [40]. However, with the presented data we show that these appeasement gestures do only partly (i.e. during AA situations) help to explain how empathic observers experience embarrassment for others' flaws or mishaps. We demonstrated that (i) even for situations in which protagonists are unaware of the embarrassing situation (AU) and protagonists behave intentionally (IA, IU), empathic observers nevertheless experience vicarious embarrassment; (ii) the level of individual differences in trait empathy is correlated with this experience; and (iii) the affective components of the 'pain matrix' are involved in this process. As noted by Tangney et al. [41], vicarious forms of social emotions substantially help to integrate social psychological research on interpersonal relations, social identity, group, and inter-group processes with cognitive and affective research.

## Supporting Information

File S1 **Pilot Studies I & II.**
(DOC)

File S2 **Supplementary methods, Study 1.**
(DOC)

File S3 **Supplementary stimulus material, Study 2.**
(DOC)

## References

[1] Keltner D, Buswell BN (1997). Embarrassment: Its distinct form and appeasement functions.. Psychological Bulletin.

[2] Miller RS, Tangney JP (1994). Differentiating embarrassment and shame.. Journal of Social and Clinical Psychology.

[3] Miller RS (1996). Embarrassment: Poise and peril in everyday life..

[4] Miller RS (1987). Empathic embarrassment: Situational and personal determinants of reactions to the embarrassment of another.. Journal of Personality and Social Psychology.

[5] Shearn D, Spellman L, Meirick J, Stryker K (1999). Empathic blushing in friends and strangers.. Motivation & Emotion.

[6] Marcus DK, Wilson JR, Miller RS (1996). Are Perceptions of Emotion in the Eye of the Beholder? A Social Relations Analysis of Judgments of Embarrassment.. Pers Soc Psychol Bull.

[7] Derbyshire SWG (2000). Exploring the pain neuromatrix.. Curr Rev Pain.

[8] Price DD (2000). Psychological and neural mechanisms of the affective dimension of pain.. Science.

[9] Singer T, Lamm C (2009). The social neuroscience of empathy.. Ann N Y Acad Sci.

[10] Lamm C, Meltzoff AN, Decety J (2009). How do we empathize with someone who is not like us? A functional magnetic resonance imaging study.. J Cogn Neurosci.

[11] Jackson PL, Brunet E, Meltzoff AN, Decety J (2006). Empathy examined through the neural mechanisms involved in imagining how I feel versus how you feel pain.. Neuropsychologia.

[12] Jackson PL, Meltzoff AN, Decety J (2005). How do we perceive the pain of others? A window into the neural processes involved in empathy.. Neuroimage.

[13] Singer T, Seymour B, O'Doherty J, Kaube H, Dolan RJ (2004). Empathy for pain involves the affective but not sensory components of pain.. Science.

[14] Jackson PL, Rainville P, Decety J (2006). To what extent do we share the pain of others? Insight from the neural bases of pain empathy.. Pain.

[15] Lamm C, Nusbaum HC, Meltzoff AN, Decety J (2007). What are you feeling? Using functional magnetic resonance imaging to assess the modulation of sensory and affective responses during empathy for pain.. PLoS One.

[16] Saarela MV, Hlushchuk Y, Williams AC, Schurmann M, Kalso E (2007). The compassionate brain: humans detect intensity of pain from another's face.. Cereb Cortex.

[17] Hein G, Singer T (2008). I feel how you feel but not always: the empathic brain and its modulation.. Curr Opin Neurobiol.

[18] Akitsuki Y, Decety J (2009). Social context and perceived agency affects empathy for pain: an event-related fMRI investigation.. Neuroimage.

[19] Immordino-Yang MH, McColl A, Damasio H, Damasio A (2009). Neural correlates of admiration and compassion.. Proc Natl Acad Sci U S A.

[20] Lamm C, Singer T (2010). The role of anterior insular cortex in social emotions.. Brain Structure and Function.

[21] Takahashi H, Yahata N, Koeda M, Matsuda T, Asai K (2004). Brain activation associated with evaluative processes of guilt and embarrassment: an fMRI study.. Neuroimage.

[22] Berthoz S, Armony JL, Blair RJ, Dolan RJ (2002). An fMRI study of intentional and unintentional (embarrassing) violations of social norms.. Brain.

[23] Berthoz S, Grezes J, Armony JL, Passingham RE, Dolan RJ (2006). Affective response to one's own moral violations.. Neuroimage.

[24] Epley N, Caruso EM, Markman KD, Klein WMP, Suhr JA (2009). Perspective taking: Misstepping into others' shoes.. Handbook of imagination and mental simulation.

[25] Epley N, Keysar B, Van Boven L, Gilovich T (2004). Perspective taking as egocentric anchoring and adjustment.. Journal of Personality and Social Psychology.

[26] Tangney JP, Miller RS, Flicker L, Barlow DH (1996). Are shame, guilt, and embarrassment distinct emotions?. Journal of Personality and Social Psychology.

[27] Leibetseder M, Laireiter A-R, Köller T (2007). Structural analysis of the E-scale.. Personality and Individual Differences.

[28] Davis MH (1983). Measuring individual differences in empathy: Evidence for a multidimensional approach.. Journal of Personality and Social Psychology.

[29] Stocks EL, Lishner DA, Waits BL, Downum EM (2011). I'm Embarrassed for You: The Effect of Valuing and Perspective Taking on Empathic Embarrassment and Empathic Concern.. Journal of Applied Social Psychology.

[30] Lamm C, Porges EC, Cacioppo JT, Decety J (2008). Perspective taking is associated with specific facial responses during empathy for pain.. Brain Res.

[31] Singer T, Seymour B, O'Doherty JP, Stephan KE, Dolan RJ (2006). Empathic neural responses are modulated by the perceived fairness of others.. Nature.

[32] Cheng Y, Lin CP, Liu HL, Hsu YY, Lim KE (2007). Expertise modulates the perception of pain in others.. Curr Biol.

[33] Lieberman MD (2007). Social cognitive neuroscience: a review of core processes.. Annu Rev Psychol.

[34] Keltner D, Young R, Buswell BN (1997). Appeasement in human emotion, personality, and social practice.. Aggressive Behavior.

[35] Goffman E (1955). On face-work; An analysis of ritual elements in social interaction.. Psychiatry.

[36] Modigliani A (1971). Embarrassment, facework, and eye contact: Testing a theory of embarrassment.. Journal of Personality and Social Psychology.

[37] Buss AH, Iscoe I, Buss EH (1979). The development of embarrassment.. Journal of Psychology.

[38] Edelmann RJ, Hampson SE (1981). Embarrassment in dyadic interaction.. Social Behavior and Personality.

[39] Semin GR, Manstead ASR (1981). The beholder beheld: A study of social emotionality.. European Journal of Social Psychology.

[40] Semin GR, Manstead ASR (1982). The social implication of embarrassment displays and restitution behaviour.. European Journal of Social Psychology.

[41] Tangney JP, Stuewig J, Mashek DJ (2007). Moral emotions and moral behavior.. Annual Review of Psychology.

