# Supplementary material for: Your Flaws Are My Pain: Linking Empathy To Vicarious Embarrassment
Source: PLoS One. 2011 Apr 13;6(4):e18675. doi: 10.1371/journal.pone.0018675 (PMC3076433; doi:10.1371/journal.pone.0018675)
Supplement: File S1 — Pilot Studies I & II. (DOC) [file pone.0018675.s001.doc]

**Supplementary Material I**

**Your Flaws Are My Pain: Linking Empathy To Vicarious Embarrassment**

**Pilot Studies I & II**

**Stimulus Material Generation**

Two pilot studies were conducted in order to construct and select vignettes which display persons in public situations violating social norms and etiquettes. The stimulus material for the following studies varies regarding the underlying dimensions of "*intentionality*" and "*awareness*" and cluster into four distinctive categories. Additionally, the vignettes reliably evoke vicarious embarrassment in the observer and are only weakly related to other emotions such as disgust, pity, and anger.

**Methods**

**Stimulus Material**

All situational vignettes were in a standardized format. They were generated in the form of simple sentences that describe common public situations. Sentences always began with the specification of the *observer's* perspective to frame the context in which the situation would take place, for example "*You* are in a bus". The beginning of the vignettes only varied with respect to the location (e.g., bus, theatre, school etc.). Following the specification of the context the observer was asked to imagine him- or herself as experiencing the actual scenario, for example "an incoming *passenger's* zipper is widely open". In this situation the *passenger* is the protagonist for whom the observer may or may not experience vicarious embarrassment. All vignettes were designed such that they fulfilled the following criteria: (1) public scenario (other observers had to be imaginable in close vicinity); (2) incidental character (no association between observer and protagonist should be assumed); (3) counterbalanced sex of the protagonist with 30% of the vignettes introducing a neutral protagonist (e.g., "someone", "a person"); (4) protagonists were always single persons (with two exceptions were a couple was used instead).

All but 22 neutral vignettes were developed to display violations of normative standards systematically manipulating the apparent "*intentionality*" and "*awareness*" of the protagonist's behavior. Overall, 91 vignettes were generated (23 for **AA**, 20 for **AU**, 24 for **IA**, 24 for **IU**). The locations specified in the vignettes were standardized and equally distributed across the four categories.

**Participants**

***Pilot Study 1.*** Sixty-four German speaking participants followed a call via e-mail to take part in a short study to evaluate different aspects of social situations. Participants were informed about the anonymity of their responses and had the option to participate in a draw of three prices of 20 Euros. Forty-six participants (71.9%) were female. The mean age was 26.19 (*SD* = 4.33) years. The largest group of participants were students (*N* = 45; 70.3%), followed by white-collar workers (*N* = 13; 20.3%) and others (*N* = 3; 4.7%).

***Pilot Study 2.*** Participants were recruited via e-mail distribution lists of student representative bodies at various German universities. Participants took part voluntarily and were informed about the anonymity of their responses. After completion of the questionnaire participants could leave their e-mail address and take part in a draw of five prices of 50 Euros. Only completed questionnaires were kept for further analyses. Six-hundred and forty-five participants followed the link provided in the e-mail of whom 295 participants (45.7%) followed the survey and completed all questions. These were 205 (69.5%) women and 90 (30.5%) men with a mean age of 24.49 (*SD* = 5.03) years. On average participants had been in education for 15.54 (*SD* = 4.25) years.

**Procedure**

***Pilot Study 1.*** The study was conducted using an online survey ([www.limesurvey.org](http://www.limesurvey.org/); version 1.72). After a welcome note, detailed rating instructions were given to the participants. Two trial situations were included in the introduction to illustrate the rating procedure. In the main part, each vignette was presented at the top of a separate web-page, and participants were asked to rate the situation using five questions.

The first two questions addressed the underlying dimensions "*intentionality*" and "*awareness*". Participants were asked whether they believed that the protagonist displayed the behavior intentionally (*"Do you assume that the person intentionally shows this behavior at that specific moment?"*) and whether they believed that the protagonist was aware that the displayed behavior may elicit vicarious embarrassment reactions in an observer *("This situation might be seen as being embarrassing for that person. Do you believe that the person is aware of this at that specific moment?").^1^* Responses were given on scales ranging from 1 (*not at all*) to 7 (*completely*). The next three questions asked participants about their personal emotions of disgust, pity, and anger while observing these situations. Emotional responses were indicated on the same seven-point scale. Repeated processing of the same questionnaire was eliminated through automatic IP-detection.

***Pilot Study 2.*** After a welcome message with introductory text the rating procedure for the questionnaire was explained with two trial situations. In the main part, the constructed vignettes were rated, with 10 vignettes presented at one webpage. Questionnaires were completed in about 30 minutes.

After first analyses of results in Pilot Study 1, 11 vignettes were excluded due to obvious misfit from the suggested a priory classification. For each of the remaining 102 vignettes, participants were asked to rate their personal vicarious embarrassment experiences while imagining the observation of the depicted situation. The instruction focused on the participants' reaction when observing the situation: "*Imagine* ***you*** *are observing the person in the situation. Are* ***you*** *feeling embarrassed for this person? If yes, how intense is this feeling?*" To highlight the personal perspective on the situation the relevant person (the observer) was highlighted in bold and the observed person was underlined.

**Results and Discussion Pilot Studies 1 and 2**

**Distribution of Situations According to the Dimensions "Intentionality" and "Awareness"**

Ratings from Pilot Study 1 were averaged across participants and linear discriminant analyses (LDA) were conducted. Mean ratings of "*intentionality*" and "*awareness*" were entered in the linear equations to classify the constructed situations according to the corresponding four categories. With the maximum of two discriminant functions overall classification was accurate, with 86.8% (*N* = 79) of the vignettes being correctly classified. These results indicate that the intended manipulation of the "*intentionality*" and "*awareness*" of the acting person was correctly evaluated by uninvolved others. Figure 1 illustrates the dimensional space of all examined vignettes.

**---------------------------**

**Figure 1 about here**

**---------------------------**

**Vignette Selection**

In order to combine the best fitting situations for further studies, vignettes were selected based on consideration of ratings in Pilot Study 1 and Pilot Study 2. In a first step, all vignettes that did not fit the intended class of situation based on attributed "*intentionality*" and "*awareness*" were discarded. In a second step, all vignettes were evaluated with respect to their elicitation of vicarious embarrassment. Three raters independently selected vignettes based on these two steps. Their selections were then combined to constitute the corpus of the vignette battery to be applied in the following studies. A total of 52 vignettes were deemed useful in assessing vicarious embarrassment on all four categories.

**Characteristics of Selected Situations**

Detailed information on mean values and standard deviations of attributed intentionality, awareness and elicited anger, disgust, and pity across the selected situations are given in Table 1. Regarding evaluations of intentionality, there was a clear distinction between the low-intentionality categories AA and AU (*M* = 1.13, *SD* = 0.15, and *M* = 1.31, *SD* = 0.31) and the high-intentionality categories IA and IU (*M* = 5.32, *SD* = 0.69, and *M* = 6.10, *SD* = 0.40). Regarding evaluations of awareness to the protagonist of the potentially embarrassing nature of the situation, the high-awareness categories AA and IA received higher ratings (*M* = 6.47, *SD* = 0.26, and *M* = 3.80, *SD* 0.65) than the low-awareness categories AU and IU (*M* = 1.81, *SD* = 0.17, and *M* = 2.49, *SD* = 0.59). However, across situations the magnitude of the difference between the categories IA and IU (*d* = 2.04) was substantially smaller than that of the difference between the categories AA and AU (*d* = 20.54). Figure 1 illustrates the distribution of selected (large symbols) and unselected (small symbols) situational vignettes according to their ratings on the dimensions "*intentionality*" and "*awareness*" as well as their individual distances to the group centroids.

---------------------------

**Table 1 about here**

---------------------------

Mean intensity patterns of the three assessed emotions differed across categories: On average pity was most strongly elicited by the (low-intentionality) AA and AU situations (*M* = 4.32, *SD* = 1.45, and *M* = 3.48, *SD* = 0.98), but less so by the (high-intentionality) IA and IU situations (*M* = 1.99, *SD* = 0.64, and *M* = 2.13, *SD* = 0.77). Thus, it was more related to unintentional mishaps. Intensity ratings of anger were relatively low overall, with the exception of IA situations (*M* = 3.36, *SD* = 1.55). It is straightforward that participants did not blame unfortunate protagonists (AA and AU) or persons not aware of the implications of their behavior (IU). Disgust was moderate in all four categories, with slightly higher ratings for AU and IA vignettes (*M* = 2.81, *SD* = 1.38, and *M* = 2.26, *SD* = 1.12). It may be more closely related to the content of the situations (e.g., loss of control over the body, shortcomings in physical appearance). Overall, standard deviations and min and max values indicate substantial variability across the situations within each category which are most likely explained through idiosyncratic characteristics of the constructed situations. However, on average the emotions pity, disgust, and anger showed plausible coherencies to the characteristics of the situation.

**---------------------------**

**Table 2 about here**

**---------------------------**

Descriptive statistics of the vicarious embarrassment experience ratings of Pilot Study 2 are displayed in Table II. After item selection the averaged responses toward vignettes of each category were close to the scale mean. However, it is notable that even after selection of best fitting situations the vicarious embarrassment ratings decreased from AA (*M* = 3.90, *SD* = 0.35) to IU (*M* = 3.10, *SD* = 0.57) situations. Averaged responses to each situation showed relatively low standard deviations within each category indicating relatively homogeneous item selections (Table III). Between subjects, the applied selection procedure allowed for computing highly reliable averages with internal consistency values for each category (Cronbach’s alpha; AA = .95; AU = .94; IA = .91; IU = .91).

Both Pilot Studies provided the basis for the following investigations of vicarious embarrassment. Pilot Study 1 demonstrated that two dimensions – the *intentionality* of a behavior attributed to a protagonist (i.e. the observed person) and the attributed *awareness* of the protagonist of the embarrassing nature of the situation – allow the classification of constructed vignettes into four largely exclusive categories. Pilot Study 2 showed that the situational vignettes do elicit vicarious embarrassment experiences in observers across all four proposed categories with highly consistent averages for each category. Compared to the three other examined emotions (pity, disgust, and anger) situations were more likely to elicit vicarious embarrassment with less dependency on idiosyncratic characteristics of single situations as indicated by the small standard deviations of ratings across situations within each category.

Table 1

*Means and Standard Deviations of Ratings for the Selected Vignettes in Pilot Study 1*

|  |  | Awareness | |  | Intention-ality | |  | Pity | |  | Anger | |  | Disgust | |
| --- | --- | --- | --- | --- | --- | --- | --- | --- | --- | --- | --- | --- | --- | --- | --- |
|  |  | *M* | (*SD*) |  | *M* | (*SD*) |  | *M* | (*SD*) |  | *M* | (*SD*) |  | *M* | (*SD*) |
| AA **(11)** | |  |  |  |  |  |  |  |  |  |  |  |  |  |  |
|  | Min | 5.90 | (1.59) |  | 1.00 | (0.00) |  | 3.60 | (1.70) |  | 1.00 | (0.00) |  | 1.00 | (0.00) |
|  | **Average** | **6.47** | **(0.26)** |  | **1.13** | **(0.15)** |  | **5.49** | **(0.78)** |  | **1.44** | **(0.39)** |  | **1.23** | **(0.30)** |
|  | Max | 6.82 | (0.59) |  | 1.55 | (1.00) |  | 6.35 | (0.88) |  | 2.08 | (1.66) |  | 1.96 | (1.29) |
| AU **(10)** | |  |  |  |  |  |  |  |  |  |  |  |  |  |  |
|  | Min | 1.56 | (1.36) |  | 1.04 | (0.20) |  | 2.91 | (1.66) |  | 1.04 | (0.20) |  | 1.27 | (0.70) |
|  | **Average** | **1.81** | **(0.17)** |  | **1.31** | **(0.31)** |  | **3.97** | **(0.81)** |  | **1.29** | **(0.29)** |  | **3.01** | **(1.34)** |
|  | Max | 2.09 | (1.51) |  | 1.86 | (1.67) |  | 5.28 | (1.40) |  | 1.91 | (1.51) |  | 5.36 | (1.68) |
| IA **(10)** | |  |  |  |  |  |  |  |  |  |  |  |  |  |  |
|  | Min | 3.05 | (1.59) |  | 4.27 | (1.67) |  | 1.27 | (1.08) |  | 1.12 | (0.44) |  | 1.16 | (0.37) |
|  | **Average** | **3.80** | **(0.65)** |  | **5.32** | **(0.69)** |  | **1.94** | **(0.64)** |  | **3.04** | **(1.79)** |  | **2.42** | **(1.25)** |
|  | Max | 4.72 | (1.67) |  | 6.36 | (1.04) |  | 2.96 | (2.21) |  | 6.16 | (1.03) |  | 4.64 | (1.81) |
| IU **(10)** | |  |  |  |  |  |  |  |  |  |  |  |  |  |  |
|  | Min | 1.44 | (0.92) |  | 4.27 | (2.13) |  | 1.64 | (1.29) |  | 1.23 | (0.53) |  | 1.32 | (0.78) |
|  | **Average** | **2.49** | **(0.59)** |  | **6.10** | **(0.40)** |  | **2.54** | **(0.90)** |  | **1.95** | **(0.63)** |  | **2.06** | **(0.58)** |
|  | Max | 3.15 | (1.84) |  | 6.55 | (0.69) |  | 4.88 | (1.76) |  | 1.23 | (0.53) |  | 3.36 | (1.89) |
| Neutral **(11)** | |  |  |  |  |  |  |  |  |  |  |  |  |  |  |
|  | Min | 1.00 | (0.00) |  | 4.14 | (2.77) |  | 1.00 | (0.00) |  | 1.00 | (0.00) |  | 1.00 | (0.00) |
|  | **Average** | **1.17** | **(0.20)** |  | **5.35** | **(0.52)** |  | **1.07** | **(0.11)** |  | **1.02** | **(0.05)** |  | **1.02** | **(0.02)** |
|  | Max | 1.55 | (1.22) |  | 6.00 | (2.09) |  | 1.27 | (0.77) |  | 1.15 | (0.49) |  | 1.05 | (0.22) |
|  |  |  |  |  |  |  |  |  |  |  |  |  |  |  |  |
| *Note. Min* = Values for Lowest Scoring Vignette; *Average* = Values for the Average of all Vignettes in Each Category (printed in bold); *Max* = Values for Highest Scoring Vignette; *SD* (Min/Max) = Standard Deviation of Ratings across Subjects; *SD* (Average) = Standard Deviation of Means across Situations. | | | | | | | | | | | | | | | |

Table 2

Means and Standard Deviations of Ratings for the Selected Vignettes in Pilot Study 2

|  | # |  | *M_min_ (SD_min_)* | |  | *M_av_ (SD_av_)* | |  | | *Median* | |  | *M_max_ (SD_max_)* | |
| --- | --- | --- | --- | --- | --- | --- | --- | --- | --- | --- | --- | --- | --- | --- |
|  |  |  |  |  |  |  |  |  |  | |  |  |  |  |
| AA | 11 |  | 3.43 | (2.07) |  | **3.90** | **(0.35)** |  | **4.01** | | |  | 4.47 | (2.16) |
| AU | 10 |  | 3.08 | (1.66) |  | **3.60** | **(0.40)** |  | **3.48** | | |  | 4.12 | (1.81) |
| IA | 10 |  | 2.86 | (1.75) |  | **3.55** | **(0.43)** |  | **3.57** | | |  | 4.01 | (2.00) |
| IU | 10 |  | 2.26 | (1.73) |  | **3.10** | **(0.57)** |  | **3.19** | | |  | 4.00 | (2.12) |
| Neutral | 11 |  | 1.03 | (0.25) |  | **1.06** | **(0.02)** |  | **1.05** | | |  | 1.12 | (0.47) |
|  |  |  |  |  |  |  |  |  |  | |  |  |  |  |
| *Notes. M_av_ =* Mean for Averaged Vignettes; *M_min_ =* Mean for Single Lowest Scoring Vignette; *M_max_ =* Mean for Single Most Strongest Scoring Vignette; The Median Refers to the Distribution of Averaged Vignettes per Category; Values Referring to the Averaged Categories are Printed in Bold; *SD* (Min/Max) = Standard Deviation of Ratings across Subjects; *SD* (Average) = Standard Deviation of Means across Situations. | | | | | | | | | | | | | | |
|

**Figure 1.** Selected and Discarded Vignettes. Large Symbols Represent Selected Vignettes and their Distance to the Group Centroids. Small Symbols Represent Unselected Vignettes.

**Footnotes**

1 All materials were in German and have been translated into English for this manuscript. As in the German language the words "embarrassment" and "shame" are used nearly synonymously, the question always was framed for both social emotions.
