# Supplementary material for: Your Flaws Are My Pain: Linking Empathy To Vicarious Embarrassment
Source: PLoS One. 2011 Apr 13;6(4):e18675. doi: 10.1371/journal.pone.0018675 (PMC3076433; doi:10.1371/journal.pone.0018675)
Supplement: File S2 — Supplementary methods, Study 1. (DOC) [file pone.0018675.s002.doc]

**Supplementary Material II**

**Your Flaws Are My Pain: Linking Empathy To Vicarious Embarrassment**

**Study 1**

**Methods**

**Participants**

In order to achieve a large sample size, more than 100 student representative bodies of German universities were asked via e-mail to distribute a short introduction advert and a link to the online survey in newsgroups and e-mail distributors of their faculties. Recipients were asked to forward the e-mail. An overlap of participants between the pilot studies (see Suppl. Material I for more detailed information) and Study 1 was ruled out through IP-address controlling. Participants were offered to leave their e-mail addresses at the end to take part in a draw of five prices of 50 Euros. Overall 1110 participants began the survey; 636 (57.3%) completed the questionnaire. Of these, 17 participants (2.7%) were excluded because their response pattern indicated that they did not follow the instruction properly.

**Materials and Procedure**

The German version of the E-scale was used to assess dispositional empathy with 25 items . It was developed to measure individual differences in empathy based on an elaborate definition of the construct differentiating between empathy in fictitious and real-life situations. The E-scale allows to measure dispositional empathy as a general factor and differentiated into four specific subfactors. The subfactors result from the combination of two underlying dimensions. One dimension covers the status of the situation: fictitious situations ("sensitivity") versus real-life situations ("concern"). The other dimension comprises the mediation mode of empathy: "cognitive" versus "emotional" mediation. Cognitive mediation accentuates the perception, reflection, and change of perspective as well as the appraisal of specific social aspects, whereas emotional mediation refers to immediate affective reactions with an observed person and the appraisal of global context . The presence of a general factor indicates that all four facets are strongly interrelated. The E-scale shows good congruent and divergent validity as well as internal consistency and re-test reliability . Means for the overall empathy score (*M* = 3.56, *SD* = 0.61) were in the upper range of the scale ranging from 1 to 5.

Finally, socio-demographic information was assessed, and participants were given the opportunity to freely report situations in which they had personally experienced vicarious embarrassment when observing someone. Overall, 107 participants (30.2%) mentioned at least one situation in which they said they had experienced vicarious embarrassment.

**References**

1. Leibetseder M, Laireiter A-R, Köller T (2007) Structural analysis of the E-scale. Personality and Individual Differences 42: 547-561.
