# Supplementary material for: Your Flaws Are My Pain: Linking Empathy To Vicarious Embarrassment
Source: PLoS One. 2011 Apr 13;6(4):e18675. doi: 10.1371/journal.pone.0018675 (PMC3076433; doi:10.1371/journal.pone.0018675)
Supplement: File S3 — Supplementary stimulus material, Study 2. (DOC) [file pone.0018675.s003.doc]

**Supplementary Material III**

**Your Flaws Are My Pain: Linking Empathy To Vicarious Embarrassment**

**Study 2**

**Stimulus Material**

All drawn sketches used in this study were designed in a standardized way such that they fulfilled the following criteria: (1) they display a public scenario (other observers are present in close vicinity); (2) the observation has an incidental character (no association between observer and actor could be assumed); (3) they have counterbalanced sex of the actor with a third of the picture stories introducing a neutral actor (e.g., "someone", "a person"); (4) the actors were always single persons (with two exceptions were a couple was used instead).

Stimulus material was validated presenting all 120 picture stories (including neutral scenarios) to *N* = 82 subjects (66 female, *mean age*/*SD* = 22.42/3.25) who (i) evaluated their personal vicarious embarrassment experiences while observing the protagonists; (ii) evaluated the level of intentionality as attributed to the protagonists' actions; (iii) and finally indicated their impression about the awareness of the protagonists in their eventual plight on a scale ranging from 1 for 'not at all' to 5 for 'very much'. For each of the four types of situations ten picture stories that were rated as strongest in eliciting vicarious embarrassment were selected for the purpose of the fMRI study. Further, selected picture stories fitted into the 'intentionality' and 'awareness' dimensions as was found before (see Supplementary Material I). Ten neutral picture stories which displayed appropriate behaviors and did not elicit any vicarious embarrassment in the observers during the pilot study completed the set of stimuli that was used in the fMRI study.
